# Genotype–Phenotype Correlation in Progressive Supranuclear Palsy Syndromes: Clinical and Radiological Similarities and Specificities

**DOI:** 10.3389/fneur.2022.861585

**Published:** 2022-04-26

**Authors:** Iñigo Ruiz-Barrio, Andrea Horta-Barba, Ignacio Illán-Gala, Jaime Kulisevsky, Javier Pagonabarraga

**Affiliations:** ^1^Movement Disorders Unit, Neurology Department, Hospital de la Santa Creu i Sant Pau, Barcelona, Spain; ^2^Sant Pau Biomedical Research Institute (IIB-Sant Pau), Barcelona, Spain; ^3^Centro de Investigación en Red - Enfermedades Neurodegenerativas (CIBERNED), Madrid, Spain; ^4^Sant Pau Memory Unit, Neurology Department, Hospital de la Santa Creu I Sant Pau, Barcelona, Spain

**Keywords:** progressive supranuclear palsy, parkinsonism, genetics, phenotype, review, genotype

## Abstract

The progressive supranuclear palsy (PSP) syndrome encompasses different entities. PSP disease of sporadic origin is the most frequent presentation, but different genetic mutations can lead either to monogenic variants of PSP disease, or to other conditions with a different pathophysiology that eventually may result in PSP phenotype. PSP syndrome of monogenic origin is poorly understood due to the low prevalence and variable expressivity of some mutations. Through this review, we describe how early age of onset, family history of early dementia, parkinsonism, dystonia, or motor neuron disease among other clinical features, as well as some neuroimaging signatures, may be the important clues to suspect PSP syndrome of monogenic origin. In addition, a diagnostic algorithm is proposed that may be useful to guide the genetic diagnosis once there is clinical suspicion of a monogenic PSP syndrome.

## Introduction

Progressive supranuclear palsy (PSP) syndrome is characterized by a progressive condition with the following cardinal features: parkinsonism, falls, and supranuclear gaze palsy. Most PSP syndromes are due to PSP disease, which mainly occurs as a sporadic condition. Additionally, there are other diseases that have been linked to PSP syndrome. Both PSP disease and PSP syndrome can have either a sporadic or monogenic origin. We have named those PSP phenotypes with a monogenic cause as genetic PSP syndrome (GPSPS). Since the clinical concept of PSP has evolved over time, the spectrum with its different phenotypic variants has recently been broadened, identifying additional phenotypes to the classical Richardson syndrome (PSP-RS), such as PSP with predominant parkinsonism (PSP-P), PSP with progressive gait freezing (PSP-PGF), PSP with predominant frontal presentation (PSP-F), PSP with predominant oculomotor dysfunction (PSP-OM), PSP with predominant speech/language disorder (PSP-SL), PSP with predominant postural instability (PSP-I), or PSP with predominant corticobasal syndrome (PSP-CBS) (1). This also opens the door to retrospectively include a greater number of cases in which, for a similar phenotype, a genetic cause has been suggested. The clinical features to differentiate sporadic from genetic conditions have been scarcely reported. In some cases, the genetic role has been well defined, but for many others, the association remains unclear, mainly due to the low number of cases reported and the lack of studies analyzing the role of each genetic mutation. A positive family history, early age of onset, and some neuroimaging features have been described as the factors that may raise suspicion of GPSPS (1–4). The aim of this review was to summarize the clinical features of the GPSPS cases reported in the literature, their associated neuroimaging findings, and those factors leading to a suspicion of a monogenic condition.

## Methods

We performed a literature review using the PubMed database during the month of December 2021. The search was restricted to the articles published in English, no date restriction was applied, and it was conducted in two steps. First, we applied the following search criteria, filtered by “review,” “systematic review,” and “meta-analysis” article types: (“Progressive supranuclear palsy” OR “PSP” OR “PSP phenotype” OR “PSP like” OR “PSP mimic^*^”) AND (“genetics^*^”[TIAB]). The result was 54 articles, of which 37 were excluded based on the title, 6 were excluded after reading the abstract, and another 6 after reading the text. The main genes involved in monogenic diseases with PSP phenotype were obtained from the remaining 5 articles: microtubule-associated protein tau gene (*MAPT*), C9orf72- SMCR8 complex subunit gene (*C9orf72*), ATPase cation transporting 13A2 gene (*ATP13A2*), NPC intracellular cholesterol transporter 1 gene (*NPC1*), NPC intracellular cholesterol transporter 2 gene (*NPC2*), granulin precursor (*GRN*), synaptojanin 1 (*SYNJ1*), dynactin subunit 1 (*DCTN1*), prion protein (*PRNP*), ataxin 3 (*ATX3*), glucosylceramidase beta (*GBA*), atrophin 1 (*ATN1*), leucine-rich repeat kinase 2 (*LRRK2*), TANK-binding kinase 1 (*TBK1*), and TAR DNA-binding protein (*TARDBP*). In a second step, a search was conducted for each gene and/or mutation we found, using the following criteria and removing filters by article type: (“Progressive supranuclear palsy” OR “PSP” OR “PSP phenotype” OR “PSP like” OR “PSP mimic^*^”) AND (“R5L” OR “L284R” OR “S285R” OR “N296” OR “P301L” OR “G303V” OR “S305S” OR “IVS10” OR “*LRRK2*” OR “*PGRN*” OR “*GRN*” OR “*DCTN1*” OR “*C9orf72*” OR “*SYNJ1*” OR “*TARDBP*” OR “*ATP13A2*” OR “*NPC1*” OR “*NPC2*” OR “*GBA*” OR “*PRNP*” OR “*ATXN3*”). We obtained 179 articles, of which 140 were excluded after reviewing titles and abstracts. After reviewing the text of the remaining 39, 36 new articles were added based on the bibliographic references, making a total of 75 articles. The clinical data and its chronology were reviewed in detail, and the PSP 2017 criteria were applied retrospectively to the cases reported in the articles, without considering the following exclusion criteria: age at onset, sporadic occurrence, and motor neuron disease (1). Those with a diagnosis of suspected, possible, or probable for PSP were included in the review (Supplementary Material).

## Genotype–Phenotype Correlation for PSP Syndromes

The main genes where mutations can lead to GPSPS are *MAPT, C9orf72, ATP13A2, NPC1/2, GRN, SYNJ1, DCTN1*, and *PRNP* (Table 1).

**Table 1:** Genes commonly associated with PSP phenotype of monogenic origin.

**Gene**	**Age at onset**	**Phenotype at onset**	**Additional features**	**Neuroimaging**	
MAPT	46.3	PSP-P and PSP-F	Family history Visual grasping	MRI PET	Mild and diffuse brain atrophy (PSP-P/RS) Symmetrical atrophy of anterior temporal lobe greater than frontal lobe, with spared cerebellum (PSP-F) Frontal reduced metabolism
C9orf72	64	PSP-RS and PSP-F	Family history Hallucinations MND	MRI PET	Symmetrical atrophy of frontal predominant with cerebellar involvement. Bilateral frontal and temporal hypometabolism
ATP13A2	14.9	PSP-P and PSP-RS	Facial-faucial-finger mini-myoclonus	MRI PET DAT	Diffuse cerebellar atrophy with cerebellar involvement Bilateral striatal hypometabolism Nigrostriatal dopaminergic dysfunction.
NPC1/2	≥15	PSP-F and PSP-OM	Refractory schizophrenia-like Ataxia Segmental dystonia Leg spasticity	MRI	Frontal and corpus callosum atrophy (PSP-F) Brainstem and cerebellar atrophy (PSP-OM)
GRN	60	PSP-F and PSP-SL	Family history Visual hallucinations Episodic memory impairment	MRI	Asymmetrical frontotemporal and parietal atrophy.
SYNJ1	24.7	PSP-P	Developmental delay Levodopa induced dyskinesias	MRI PET DAT	Normal or cortical and midbrain atrophy Cortical and caudate hypometabolism Nigrostriatal dopaminergic dysfunction.
DCTN1	59.5	PSP-P and PSP-F	Family history	MRI SPECT	Mild diffuse or frontoparietal atrophy with brainstem involvement Frontal hypoperfusion
PRNP	52.9	PSP-P and PSP-F	Cerebellar ataxia Nystagmus	MRI	Normal, cortical ribbon, iron deposition in the basal ganglia and/or cerebellar atrophy.

*PET: FDG-PET; DAT: DAT-SPECT*.

### *MAPT* Gene-Related PSP Syndrome

Microtubule-associated protein tau gene mutations have been associated with different neurodegenerative diseases that share pathological changes with tau protein accumulation. Specifically, it has been associated with Alzheimer's disease, corticobasal degeneration, PSP, Pick disease, and argyrophilic grain disease. Between 0.6 and 14.3% of total PSP disease cases are due to *MAPT* mutations (3). Among GPSPS, mutations related to the *MAPT* gene appear to be the most frequent cause. The pattern of inheritance is autosomal dominant, penetrance is frequently complete, and there is usually variable expressivity, with different phenotypes observed for the same mutation. The most frequently reported mutation is IVS10+3 (35%). While dementia is generally a frequent feature in *MAPT* mutations (5, 6), among GPSPS, 51.1% presented with PSP-parkinsonism (PSP-P, 20–24%) or PSP-RS (11–18%) motor features, and 41.9% had frontal predominant cognitive symptoms at onset (PSP-F). Mutations related to a motor phenotype onset are R5L, L284R, S285R, delN296, N296N, and G303V, and those associated with cognitive symptoms at diagnosis are P301L, S305S, and IVS10+3. The mean age at presentation is 46.3 years (range 32–63 years). Upper and lower motor neuron involvement or denervation was observed in some cases for P301L and IVA10+3 mutations. As a differential clinical feature, visual grasping has been observed in N296N mutations, a sign associated with medial frontal lobe dysfunction leading to forced fixation and dependency of gaze on objects brought into the patient's field of view (7). On magnetic resonance imaging (MRI), *MAPT* mutations are generally associated with predominant and symmetric atrophy in the frontotemporal lobes, with paramagnetic accumulation in midbrain nuclei and white matter changes. Anterior temporal atrophy is symmetric and more marked than orbitofrontal and lateral prefrontal atrophy. Mutations in the splicing region usually lead to medial temporal lobe involvement and mutations in the coding regions affect the lateral temporal lobe. In ^18^fluorodeoxyglucose positron emission tomography (FDG-PET), the most common pattern is bilateral temporal hypometabolism (4). For GPSPS, this pattern of frontotemporal atrophy was found on computed tomography (CT) and MRI, as well as frontal hypometabolism on FDG-PET, but only for PSP-F presentations. MRI of cases with PSP-P showed mild and diffuse brain atrophy as the main pattern (8–25). In patients with a clinical diagnosis of PSP, *in vivo* hippocampal, basal ganglia, and midbrain retention of 18F-AV-1451 tau PET have been seen. Patients with *MAPT* S305N (SUVRs) and P301L mutations have shown lower levels of tau PET-specific standardized uptake value ratios (SUVRs) at the temporal poles than patients with mutations outside exon 10, and P301L mutations have shown lower levels of tau PET in the white matter (26, 27).

### *C9orf72* Gene-Related PSP Syndrome

The GGGGCC hexanucleotide repeat expansion in the non-coding region of the first exon leads to haploinsufficiency (28) and additional unclear factors (29) that lead to neuronal dysfunction. This expansion is a frequent cause of amyotrophic lateral sclerosis (ALS) and frontotemporal dementia (FTD) (30, 31). The clinical spectrum has expanded in the last years, including genotype–phenotype associations to other neurodegenerative diseases such as PSP, corticobasal syndrome (CBS), Parkinson's disease (PD), multiple system atrophy, Huntington disease-like syndrome, and Creutzfeldt–Jakob disease (CJD) (31–34). Yet, its exact role in these neurodegenerative diseases remains largely unknown (35, 36). Up to 35% of patients with the *C9orf72* gene expansion have parkinsonism (6) and 7% of patients with PSP syndrome, and a positive family history carries the *C9orf72* gene expansion, representing the second-most frequent genetic mutation related to GPSPS after *MAPT*. For those patients with >30 repeats and PSP syndrome, the mean age at presentation was 64 years (range 40–79), and 12% of patients had a family history of neurodegenerative disease. The PSP-RS phenotype was present in the majority of these cases, and PSP-F was the second-most frequent phenotype. Family history following an autosomal dominant inheritance pattern, frequent hallucinations, and motor neuron disease may be differential findings in this entity. As in *MAPT* mutations, a symmetric atrophy of the anterior temporal lobe has been described, but it differs from *MAPT* in greater involvement of the cerebellum in combination with atrophy of the medial prefrontal, dorsolateral, and orbitofrontal cortex (4). Both cortical and midbrain atrophy as well as frontotemporal-predominant atrophy were observed in patients with GPSPS (37–40).

### *ATP13A2* Gene-Related PSP Syndrome

Kufor–Rakeb syndrome is an autosomal recessive disease due to *ATP13A2* gene mutations, which has been related to levodopa-responsive juvenile parkinsonism with associated dyskinesias, supranuclear vertical gaze palsy, and prominent psychiatric symptoms (41). The mean age at the onset of the disease is 14.9 years (range 7.5–22 years). Although psychiatric symptoms are frequent, diagnosis is based on severe motor symptoms at onset, with PSP-P and PSP-RS being the most frequent phenotypes. A distinctive clinical feature present in all cases is the development of clinically relevant facial-faucial-finger mini-myoclonus, which are jerky movements in the perioral area, tongue, and fingers. Neuroimaging shows diffuse cerebral atrophy with cerebellar involvement in most patients, unrelated to the disease progression. Then, one patient showed bilateral striatal hypometabolism in FDG-PET and dopamine transporter single-photon emission computed tomography (DAT-SPECT) studies, consistent with nigrostriatal dopaminergic dysfunction (42, 43).

### *NPC1/2* Gene-Related PSP Syndrome

Niemann–Pick disease type C is a lysosomal disorder of autosomal recessive inheritance due to mutations in the *NPC1* or *NPC2* genes. In infantile forms, ataxia, dystonia, bulbar symptoms, epilepsy, gelastic cataplexy, and developmental delay predominate. Clinical symptoms in the adult onset presentations have a slower progression, but are characterized by severe cognitive and psychiatric disturbances (schizophrenia-like symptoms often refractory to treatment), ataxia, segmental dystonia with predominant facial and bibrachial involvement, vertical supranuclear gaze palsy (especially downward), and occasional bilateral leg spasticity (6, 44). Among the adolescent–adult cases reported that met clinical criteria for PSP, the most frequent phenotype at onset was PSP-F. These cases showed frontal predominant cortical atrophy on MRI, sometimes associated with corpus callosum atrophy. Those with predominant oculomotor symptoms were more likely to have brainstem and cerebellar atrophy, with relative sparing of cortical and subcortical areas (45). When patients with a PSP phenotype and additional symptoms that raise the suspicion of Niemann–Pick type C, screening with filipin staining is a good approach prior to genetic study.

### *GRN* Gene-Related PSP Syndrome

Granulin precursor gene mutations have been mainly associated with Alzheimer's disease, FTD, CBS, progressive non-fluent aphasia, and cortical sensory symptoms (6, 46). Most mutations produce a stop codon resulting in haploinsufficiency. Mutations in the *GRN* gene, however, have been also found in patients with the PSP syndrome. The mean age at onset is around 60 years (range 63–68), and 50% of patients had a known family history of parkinsonism, PSP, or behavioral variant of frontotemporal dementia (bvFTD). Between 50 and 75% of patients with PSP syndrome and *GRN* mutation had an initial phenotype of PSP-F, and the remainder had PSP-SL and PSP-P phenotypes. As a differential clinical feature of *GRN* gene mutations, up to 25% of patients developed visual hallucinations and delusions. Orobuccal dyskinesias were common, although many reported patients had previous exposure to neuroleptics. The usual pattern of *GRN* mutations on MRI is different from *MAPT* mutations, following a distribution of asymmetric frontotemporal atrophy and additional involvement of the inferior parietal lobe. More specifically, patients with the 102delC mutation of *GRN* gene show more widespread frontal, temporal, and brainstem atrophy on MRI (47–51).

### *SYNJ1* Gene-Related PSP Syndrome

Mutations in the *SYNJ1* gene, also known as the *PARK20* gene, have been associated with monogenic parkinsonism with autosomal recessive inheritance and predominant postural instability (52), dystonia, cognitive impairment, and seizures (53). Some cases with PSP syndrome have been described, with a very early disease onset. Mean age is 24.7 years (range 21–28), with PSP-P being the most frequently reported phenotype. One case was associated with developmental delay, and in another two cases, levodopa-induced dyskinesias were frequent. Apart from one case showing cortical and midbrain atrophy, MRI has no specific abnormalities. Cortical and caudate hypometabolism was observed with FDG-PET, and DAT-SPECT showed a bilateral and severe nigrostriatal deficit (54–57).

### *DCTN1* Gene-Related PSP Syndrome

The dynactin gene, *DCTN1*, has classically been associated with Perry syndrome, which is defined by parkinsonism, psychiatric symptoms, central hypoventilation, and bodyweight loss (58). Following the discovery of the gene, the clinical spectrum associated with mutations in *DCTN1* has broadened to include FTD, motor neuron disease, and PSP syndrome (6, 59). Most reported cases (60–80%) had a PSP-P phenotype, and 20–40% had PSP-F. The mean age at presentation was 59.5 years (range 46–84), and all patients had family history of bvFTD, Perry syndrome, or PD. MRI findings were variable, ranging from mild diffuse atrophy to brainstem or predominant frontoparietal atrophy. Frontal hypoperfusion was also demonstrated in one case by SPECT (60–63).

### *PRNP* Gene-Related PSP Syndrome

Among the main genetic prion diseases due to mutations in the *PRNP* gene, genetic CJD, Gerstmann–Sträussler–Scheinker disease, and fatal familial insomnia are known. While the presentation can be heterogeneous, the most common features include dementia, psychiatric disturbances, myoclonus, ataxia, cortical blindness, extrapyramidal symptoms, and insomnia (64–66). Some families with PSP phenotype have been described, with PSP-P or PSP-F associated with initial psychiatric symptoms following PSP. The mean age at presentation was 52.9 years. All patients had ataxia and/or nystagmus. MRI showed heterogenous findings, such as non-specific white matter changes, cerebellar atrophy, basal ganglia iron deposition, and cortical ribbon hyperintense on DWI and FLAIR sequences (66–69). In patients with the PSP syndrome, a family history with autosomal dominant inheritance pattern is found.

### Other Genes Rarely Associated With PSP Syndrome

Some monogenic diseases can mimic the PSP phenotype in early stages. In these cases, PSP syndrome is usually an incidental finding, and the disease course reveals other clinical features that allow the exclusion of the diagnosis (Table 2).

**Table 2:** Genes rarely associated with PSP phenotype of monogenic origin.

**Gene**	**Age at onset**	**Phenotype at onset**	**Additional features**	**Neuroimaging**	
ATX3	40	PSP-OM	Cerebellar ataxia Nystagmus	MRI	Cerebellar atrophy
GBA	54.5	PSP-RS and PSP-CBS	Cerebellar ataxia Myoclonic epilepsy Spasticity	MRI PET	Normal Occipital-temporal-parietal hypometabolism
ATN1	30	PSP-P and PSP-OM	Cerebellar ataxia Nystagmus Myoclonus Epilepsy	NA	Thalamic and pallidal hyperintensities
LRRK2	68.6	PSP-P or PSP-RS	NA	MRI	Midbrain atrophy and mild diffuse atrophy
TBK1	62	PSP-F	NA	MRI	Midbrain atrophy
TARDBP	68,5	PSP-P	NA	NA	NA

*NA, not applicable; PET: FDG-PET*.

#### ATX3 Gene-Related PSP Syndrome

Spinocerebellar ataxia type 3 (SCA3), also known as Machado–Joseph disease, is an autosomal dominant disorder due to CAG repeat in the *ATXN3* gene that leads to cerebellar ataxia, pyramidal signs, dystonia, peripheral neuropathy, and progressive external ophthalmoplegia, although the clinical heterogeneity is the hallmark of this entity (70, 71). Subtle oculomotor disturbances such as square wave jerks or saccadic slowing are common in preclinical SCA3 and they may resemble the PSP-OM phenotype (72). In these cases, nystagmus could be a differential sign at that stage, and the progressive development of ataxia will be the key feature for the diagnosis. Progressive cerebellar atrophy on MRI, which is often already visible in early stages, can also be helpful (73).

#### GBA Gene-Related PSP Syndrome

In sphingolipid storage diseases such as Gaucher disease (GD), due to mutations of the *GBA* gene with autosomal recessive inheritance pattern, abnormal saccadic eye movements are not uncommon. In GD type 3, with childhood and adolescent onset, horizontal supranuclear gaze palsy can be seen, often in combination with ataxia, myoclonic epilepsy, spasticity, and dementia (74). GD type 1 usually presents with systemic changes (95%) such as splenomegaly, hepatomegaly, anemia, thrombocytopenia, and skeletal changes (75), and neurological symptoms are uncommon. In two GD type 1 families, however, patients with PSP-RS and PSP-CBS phenotypes and mean age of onset around 55 years have been described. MRI was unremarkable, but PET-FDG showed hypometabolism in the temporo-parieto-occipital region (76, 77).

#### ATN1 Gene-Related PSP Syndrome

Dentatorubral–pallidoluysian atrophy (DRPLA) is an inherited autosomal dominant disease due to CAG trinucleotide expansion in the *ATN1* gene. It is more common in Japanese population, and myoclonus, epilepsy, ataxia, and dementia are frequent, with disease onset during the third decade of life (78). Cases with >100 CAG repeats with PSP-OM and PSP-P phenotypes have been reported. All cases were associated with ataxia and nystagmus (79, 80).

#### LRRK2 Gene-Related PSP Syndrome

Mutations in the *LRRK2* gene are mainly associated with PD (81), but they have been rarely associated also with PSP symptoms (3). A total of four families with PSP syndrome and *LRRK2* mutations have been reported. All of them presented with PSP-P or PSP-RS. Mean age at presentation was 68.6 years. In one patient, midbrain atrophy was prominent on MRI, and in another case, there was mild diffuse atrophy (82, 83).

#### Others

A case of a family with mutation in *TBK1* gene and PSP-postural instability (PSP-PI) phenotype has been reported. She presented at 62 years of age and developed frontal cognitive symptoms. Brain MRI showed midbrain atrophy (84). A total of two cases are also known with mutation in the *TARDBP* gene and a motor phenotype of PSP-P, but no neuroimaging data were available (84, 85).

## Discussion

Currently, accepted Movement Disorder Society diagnostic criteria for PSP (MDS-PSP) have advanced in the recognition of PSP as a heterogeneous clinical entity with a varying constellation of symptoms at disease onset and differential clinical progression. Nevertheless, overlap exists between phenotypes during disease evolution, and clinicopathological studies demonstrated that the accuracy of PSP diagnosis decreases from cases fulfilling criteria of probable PSP to patients with a diagnosis of possible PSP or suggestive of PSP (86–88). Phenotypic classification has been particularly useful for retrospective studies, where up to 76% of cases showed phenotypes other than PSP-RS (89). The observed overlap between the different phenotypes over time—especially for PSP-P and PSP-RS—can also be found when MDS-PSP criteria are retrospectively applied in genetically based diseases. As we have reviewed in this article, patients with different genetic mutations develop parkinsonian symptoms, early oculomotor disturbances, postural instability, or frontal-lobe symptoms that meet criteria for a clinical diagnosis of PSP. In this review, we attempted to apply the criteria to the initial clinical presentation of different genetic conditions, since over time, the progression to PSP symptoms is quite common in most patients, as well as the appearance of other additional features that could constitute exclusion criteria. Phenotypic classification has also allowed to capture the overlap in the cognitive behavioral spectrum of PSP-F and the behavioral variant of FTD, which is observed more frequently in these genetically based diseases than in patients with sporadic PSP (6, 90).

Advances in genetics have allowed to understand better the phenotypic complexity of neurodegenerative diseases. The identification of novel mutations has disclosed overlapping genotype–phenotype correlations and relevant clinical and imaging differences with previously defined sporadic cases. The application of currently accepted clinical criteria is needed for clinical practice and research, but clinical data are enriched with a deeper knowledge on the different genetic abnormalities that lead to neuronal dysfunction. This is especially seen in the case of GPSPS and in the so-called “PSP look-alike syndromes,” where distinctive features will usually appear during the course of the disease which then preclude the diagnosis of sporadic PSP. The heterogeneous inclusion of an overlap of sporadic and genetic disorders with different etiologies in the early stages of the disease for the different degrees of probability of PSP syndrome may delay the early etiological diagnosis of disorders other than sporadic PSP disease.

**Figure 1:**
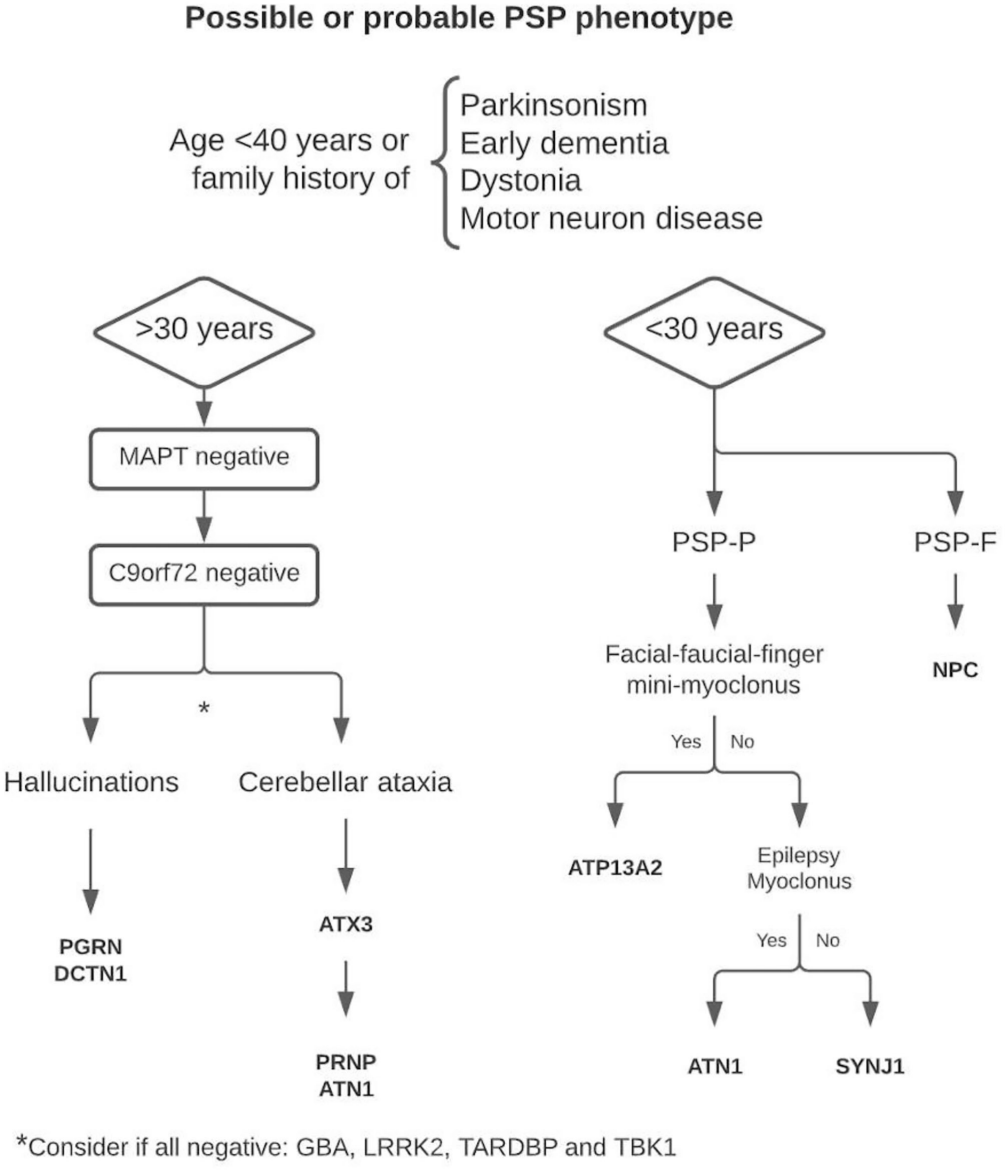
Proposed decision algorithm for genetic diagnostics.

In this review, we have shown, from a pragmatic point of view, that when “sporadic occurrence,” “age of onset,” or “motor neuron disease” criteria are not met, the entity should still be considered as a “PSP syndrome,” since it can be a fundamental clinical tool to guide the differential diagnosis in those cases without additional exclusion criteria and suspicion of genetic basis. For this purpose, we suggest a decision algorithm to facilitate early genetic diagnosis in these cases (Figure 1), which usually have a longer diagnostic delay due to their low prevalence. Early age of onset, family history of early dementia, parkinsonism, dystonia, or associated motor neuron disease are the commonly reported features that seem useful in the genetic study of patients with PSP syndrome. Based on this comprehensive review, we consider that the age of 30 years may be an optimal cutoff point to differentiate between some of the implicated genes. Because of its high frequency and phenotypic variability, we consider that in all patients with PSP syndrome with onset > 30 years of age and a suspicion of genetic origin, the *MAPT* gene should be studied first. The prevalence of *C9orf72* is considerably higher than the other mutations, so its determination is recommended in *MAPT*-negative cases regardless of clinical features. If negative, the presence of early and prominent hallucinations can be an important clue to decide the next candidate genes. The same is true for the appearance of cerebellar ataxia. Even when ataxia is not a prominent or dominant clinical feature, this symptom suggests the underlying etiology. In those cases, with onset under 30 years of age, the presence of facial-faucial-finger mini-myoclonus, epilepsy, or generalized myoclonus may be important data guiding the diagnosis.

This approach can be particularly useful when genetic panels are not available or are incomplete due to missing genes. It should, however be taken into account that due to different genetic factors such as incomplete penetrance or age-related penetrance in autosomal dominant monogenic diseases, the diagnosis can be challenging, especially in late-onset cases.

Overall, the recognition of clinical syndromes with suspected monogenic origin and a PSP syndrome may be useful in clinical practice. The recognition of larger number of cases with well-defined mutations, and their relationship with specific clinical features and neuroimaging findings, would provide insight into the research of their causal role and pathophysiological mechanisms leading to neuronal damage.

## Author Contributions

IR-B collected cases from the literature that met the established criteria. JP supervised the task and contributed to the drafting of the manuscript. II-G, AH-B, and JK made collaborative contributions to move the project forward. All authors contributed to the article and approved the submitted version.

## Conflict of Interest

The authors declare that the research was conducted in the absence of any commercial or financial relationships that could be construed as a potential conflict of interest.

## Publisher's Note

All claims expressed in this article are solely those of the authors and do not necessarily represent those of their affiliated organizations, or those of the publisher, the editors and the reviewers. Any product that may be evaluated in this article, or claim that may be made by its manufacturer, is not guaranteed or endorsed by the publisher.
