# Supplementary material for: Genotype–Phenotype Correlation in Progressive Supranuclear Palsy Syndromes: Clinical and Radiological Similarities and Specificities
Source: Front Neurol. 2022 Apr 26;13:861585. doi: 10.3389/fneur.2022.861585 (PMC9087829; doi:10.3389/fneur.2022.861585)
Supplement: Supplementary file 1 [file Table_1.DOCX]

Clinical and demographic characteristics of included PSP-ph cases

| **GENE** | **MUTATION** | **N** | **ORIGIN** | **AO** | **ONSET PHENOTYPE** | **OTHER SIGNS** | **REFERENCES** |
| --- | --- | --- | --- | --- | --- | --- | --- |
| **MAPT** | p. R5L | 1 | American | 41 | prob. PSP-RS | - | 9 |
|  | p.L284R | 3 | British  British  British | 40  48  43 | poss. PSP-OM  prob. PSP-RS/F  prob. PSP-RS/F | -  -  - | 10  10  10 |
|  | p.S285R | 3 | Dutch  Dutch  Japanese | 40  41  46 | poss. PSP-OM  prob. PSP-RS  prob. PSP-RS | L-imb dystonia  Bell’s phenomenon (grasping?) | 11  11  12 |
|  | p.delN296 | 2 | Italian  Spanish | 39  39 | prob. PSP-RS  prob. PSP-F/CBS | -  - | 13  26 |
|  | p.N296N | 3 | Japanese  Japanese  Japanese | 42  41  43 | prob. PSP-P  prob. PSP-P/RS  prob. PSP-P/RS | Oscillopsia  Visual grasping  Visual grasping | 15  12  12 |
|  | p.P301L | 4 | French  Canadian  American  Argentinean | 57  63  41  56 | prob. PSP-F  -  prob. PSP-P  prob. PSP-F/poss. PSP-SL | Fasciculations and brisk reflexes  -  Fasciculations and brisk reflexes  - | 27  18  17  16 |
|  | p.G303V | 7 | French  French  French  French  French  French  French | 37  41  37  51  39  42  32 | prob. PSP-RS  s.o. PSP-P  prob. PSP-P  s.o PSP-P  prob. PSP-P  prob. PSP-RS  prob. PSP-P | -  -  -  -  -  -  - | 20  28  28  28  28  28  28 |
|  | p.S305S | 5 | Australian  Australian  Australian  Finnish  Finnish | 55  48  54  46  40 | prob. PSP-F/poss. PSP-SL  prob. PSP-P  prob. PSP-F/poss. PSP-SL  prob. PSP-F  prob. PSP-F | -  -  -  -  - | 21  21  21  22  22 |
|  | IVS10+3 | 15 | Italian  Italian-Polish  British (?)  British (?)  British (?)  British (?)  British (?)  British (?)  British (?)  British (?)  British (?)  British (?)  British (?)  British (?)  British (?) | 50  61  50  45  42  54  45  39  50  47  52  58  58  52  48 | poss. PSP-SL  prob. PSP-F  prob. PSP-F  prob. PSP-F  prob. PSP-F  prob. PSP-F  prob. PSP-P  prob. PSP-F  prob. PSP-F  s.o. PSP-P  prob. PSP-P  s.o. PSP-F/poss. PSP-SL  prob. PSP-F  s.o. PSP-F  s.o. PSP-P | -  -  -  -  -  -  Hearing loss  Up-beating nystagmus  -  -  Denervation  Paresthesia  Denervation  -  -  -  - | 23  24  25  25  25  25  25  25  25  25  25  25  25  25  25 |
| **LRRK2** | R1441H | 1 | Cretan | 61 | s.o. PSP-P | - | 83 |
|  | p.A1413T | 1 | Filipino | 72 | prob. PSP-RS/P | - | 84 |
|  | p.G2019S | 1 | Amer. (?) | 73 | prob. PSP-RS | - | 84 |
| **PGRN** | c.813_816delCACT | 1 | Italian | 68 | s.o PSP-F | - | 48 |
|  | c.1477CT | 1 | Irish | 63 | pos. PSP-SL/s.o PSP-F | Difficulties in speech  Orobuccal dyskinesia | 49 |
|  | c.102delC | 1 | Czech | 64 | s.o PSP-F/s.o. PSP-P | Possible denervation | 50 |
|  | del SA470 | 1 | Italian | 63 | s.o PSP-P/prob. PSP-RS | - | 51 |
|  | c.720CT | 1 | Korean | - | - | - | 52 |
| **DCTN1** | G71E | 2 | French  French | 59  49 | s.o. PSP-P  prob. PSP-F/P | -  Limb dystonia | 61  61 |
|  | G71R | 1 | British(?) | 46 | s.o. PSP-F | - | 64 |
|  | p.K56R | 2 | Chinese  Taiwanese | -  - | prob. PSP-P  prob. PSP-P | -  - | 62  62 |
|  | c.G36A | 1 | - | 84 |  | - | 63 |
| **C9Orf72** | >30 GGGGCC repeat | 123  2 | -  French  Italian | 64  55  68 | prob. PSP-RS  s.o. PSP-F  prob. PSP-P/RS | -  -  - | 38  39  40 |
| **SYNJ1** | Compound c.4217_4218insC and c.4126A>G | 1 | Tunisian | 21 | prob. PSP-P/RS | - | 55 |
|  | p.Arg258Gln homozyg. | 3 | Italian  Italian  Italian | 28  28  22 | s.o PSP-P  s.o PSP-P  s.o PSP-P | Developmental delay  L-dopa induced dystonia  Early anarthria  Limb dystonia  L-dopa induced oro-mandibular and limb dyskinesia | 56  57  58 |
| **TBK1** | p.Glu643del | 1 | Turkish | 62 | s.o PSP-PI | - | 85 |
| **TARDBP** | p.A382T | 2 | Sardinian | 67  70 | s.o. PSP-P  prob. PSP-P/RS | - | 86 |
| **ATP13A2** | c.1632_1653dup22 | 4 | Jordanian | 13 | prob. PSP-P/RS | FFF mini-myoclonus | 44 |
|  | c.3176T>G  c.3253delC | 2 | Chinese | 17 | prob. PSP-P/RS | FFF mini-myoclonus  Olfactory dysfunction |  |
|  | c.2552_2553delTT | 1 | Afghan | 10 | prob. PSP-P | FFF mini-myoclonus |  |
|  | c.1103_1104insGA | 1 | Pakistani | 17 | prob. PSP-P/RS | FFF mini-myoclonus  Olfactory dysfunction |  |
|  | c.3057delC  c.130615G>A | 5 | Chilean | 13 | prob. PSP-P/RS | FFF mini-myoclonus  Olfactory dysfunction |  |
|  | c.1510G>C | 1 | Brazilian | 12 | prob. PSP-RS |  |  |
|  | c.546C>A | 1 | Japanese | 22 | prob. PSP-P/RS | FFF mini-myoclonus |  |
|  | c.2629G>A | 2 | Italian | 7.5 | prob. PSP-P | FFF mini-myoclonus |  |
|  | c.2473delCinsAA | 6 | Inuit | 19.5  - | prob. PSP-P/RS | FFF mini-myoclonus |  |
|  | c.2572C>T | 3 | Iranian | 18 | prob. PSP-P/RS | FFF mini-myoclonus |  |
| **NPC1/2** | I1061T, V39M/V39M… | - | American, British, French, Canadian, Portuguese, Italian, German | - | prob. PSP-F/poss. PSP-OM | Chorea, limb dystonia, ataxia, myoclonus  Hearing loss | 46 |
| **GBA** | N370S (SA492) | 2 | Italian  German | 41  68 | poss. PSP-CBS  prob. PSP-RS | Agraphia  - | 77  78 |
| **PRNP** | A133V  M129V | 1  1 | Indonesian  Spanish | 62  71 | prob. PSP-RS  prob. PSP-F | - | 67 |
|  | c.198S>F | 4 | American  American  American  American | 51  65  57  40 | prob. PSP-P/F  s.o. PSP-P  s.o. PSP-OM  prob. PSP-P/F | Nystagmus  Nystagmus, ataxia  Nystagmus, ataxia  Nystagmus, ataxia  Myoclonus, ataxia | 68  68  68  68 |
|  | c.200E>K | 5 | German  German  German  German  German | 48  64  59  44  48 | s.o PSP-OM  s.o PSP-OM  s.o PSP-OM  s.o PSP-OM  s.o PSP-OM | Ataxia  Ataxia  Ataxia  Ataxia  Ataxia | 69  69  69  69  69 |
| **ATN1** | >100 CAG | 3  1 | Japanese  Japanese  Japanese  Chinese | 15  13  38  48 | prob. PSP-P  s.o PSP-OM  s.o PSP-OM  prob. PSP-RS | Ptosis, ataxia, nystagmus, limb dystonia, atetosis  Ataxia, nystagmus, limb dystonia  Ataxia, nystagmus  Ataxia, dystonia, chorea | 80  80  80  49 |
| **ATXN3** | >56 CAG |  | - | 35 | prob. PSP-P/s.o PSP-OM | Ataxia, nystagmus, limb dystonia | 71, 43 |

AO = Age of onset
