# Supplementary material for: Genotype–Phenotype Correlation in Progressive Supranuclear Palsy Syndromes: Clinical and Radiological Similarities and Specificities
Source: Front Neurol. 2022 Apr 26;13:861585. doi: 10.3389/fneur.2022.861585 (PMC9087829; doi:10.3389/fneur.2022.861585)
Supplement: Supplementary file 2 [file Data_Sheet_3.docx]

Neuroimaging details of included cases with PSP-ph

| **GENE** | **MUTATION** | **N** | **NEUROIMAGE** | **REFERENCES** |
| --- | --- | --- | --- | --- |
| **MAPT** | p. R5L | 1 | MRI: cerebral atrophy and multiple areas of increased signal intensity in the white matter of both hemispheres | 9 |
|  | p.L284R | 3 | -  -  - | 10  10  10 |
|  | p.S285R | 3 | -  -  - | 11  11  12 |
|  | p.delN296 | 2 | -  MRI: mild, diffuse cerebral atrophy | 13  26 |
|  | p.N296N | 3 | -  -  - | 15  12  12 |
|  | p.P301L | 4 | MRI: mild frontal atrophy. SPECT: decreased symmetrical bifrontal metabolism.  -  CT: slight prominence of the sulci over the cerebral convexity  CT: bilateral frontal and temporal atrophy | 27  18  17  16 |
|  | p.G303V | 7 | Fluordopa F18: asymmetrical uptake  -  -  -  -  -  - | 20  28  28  28  28  28  28 |
|  | p.S305S | 5 | MRI: mild atrophy of the posterior frontal lobe  FDG-PET: bilateral frontal and temporal low metabolism  MRI: mild atrophy over the vertex  FDG-PET: asymmetric reduction in metabolism in the right frontoparietal cortices, thalamus and striatum  -  -  CT: temporal lobe atrophy | 21  21  21  22  22 |
|  | IVS10+3 | 15 | -  -  CT: marked frontal and temporal atrophy  MRI: cerebral atrophy in frontal and temporal lobes  -  -  MRI and VBM: loss of total grey matter volume and increased CSF volume. Most significant focal points of large clusters of grey matter loss occurred bilaterally in medial temporal lobe, opercular cortex and insular cortex. During follow-up: atrophy in the superior frontal and cingulate gyri, anterior temporal lobe, hippocampus, parahipocampal gyrus, frontal and anterior temporal lobes.  -  -  -  -  -  -  -  - | 23  24  25  25  25  25  25  25  25  25  25  25  25  25  25 |
| **LRRK2** | R1441H | 1 | - | 83 |
|  | p.A1413T | 1 | MRI: brain stem atrophy | 84 |
|  | p.G2019S | 1 | MRI: mild generalized atrophy | 84 |
| **PGRN** | c.813_816delCACT | 1 | MRI: enlarged perimesencephalic cisterns | 48 |
|  | c.1477CT | 1 | MRI: no frontal or temporal atrophy | 49 |
|  | c.102delC | 1 | MRI: frontal, temporal and brainstem atrophy | 50 |
|  | del SA470 | 1 |  | 51 |
|  | c.720CT | 1 | - | 52 |
| **DCTN1** | G71E | 2 | MRI: midbrain atrophy  SPECT: prefrontal superior hypoperfusion | 61  61 |
|  | G71R | 1 | MRI: mild diffuse cerebral atrophy and more pronounced focal midbrain atrophy. | 64 |
|  | p.K56R | 2 | MRI: symmetrical temporal and frontoparietal atrophy | 62  62 |
|  | c.G36A | 1 | - | 63 |
| **C9Orf72** | >30 GGGGCC  repeat | 123  3 | -  MRI: moderate diffuse cortical and mesencephalic atrophy  MRI: frontal and anterior temporal region atrophy | 38  39  40 |
| **SYNJ1** | Compound c.4217_4218insC and c.4126A>G  p.Arg258Gln homozyg. | 3 | MRI: normal  MRI: normal  DAT-Scan: severe, bilateral nigrostriatal dopaminergic deficit  18-FDG PET: cortical and caudate mild hypometabolism  MRI: diffuse cortical atrophy, hyperintensity of hippocampi, thinning midbrain quadrigeminal plate  18-FDG PET: cortical hypometabolism | 55 |
| **TBK1** | p.Glu643del |  | CT: mesencephalic atrophy. The anteroposterior midbrain diameter was 14.3 mm. Cortical atrophy, pronounced in the frontal cortex. | 56  57  58 |
| **TARDBP** | p.A382T | 2 | -  - | 85 |
| **ATP13A2** | c.1632_1653dup22 | 4 | MRI: brain atrophy involving cerebral hemispheres and the cerebellum.  18FDG-PET: reduced bilateral tracer uptake in the striatum  SPECT: reduced bilateral tracer binding in the striatum or caudate and putamen | 86  44 |
|  | c.3176T>G  c.3253delC | 2 |  |  |
|  | c.2552_2553delTT | 1 |  |  |
|  | c.1103_1104insGA | 1 |  |  |
|  | c.3057delC  c.130615G>A | 5 |  |  |
|  | c.1510G>C | 1 |  |  |
|  | c.546C>A | 1 |  |  |
|  | c.2629G>A | 2 |  |  |
|  | c.2473delCinsAA | 6 |  |  |
|  | c.2572C>T | 3 |  |  |
| **NPC1/2** | I1061T*^1^, V39M/V39M… |  | MRI:  - Psychiatric onset: cortical atrophy predominating in the frontal lobes, sometimes with corpus callosum atrophy.  - Motor onset: brainstem and cerebellas atrophy with relative sparing of the cortical and subcortical areas | 46 |
| **GBA** | N370S (SA492) | 2 | MRI: unremarkable  18F-FDG PET: bilateral occipito-temporo-parietal hypometabolism with left predominance  - | 77  78 |
| **PRNP** | A133V | 1 | MRI: nonspecific periventricular white matter changes | 67 |
|  | c.198S>F | 4 | MRI: cerebellar atrophy and iron deposition in basal ganglia.  -  -  MRI: decreased signal in the basal ganglia and cerebellar atrophy. | 68  68  68  68 |
|  | c.200E>K | 5 | -  -  -  -  - | 69  69  69  69  69 |
|  | >100 CAG | 5 | -  -  -  -  Nonspecific mild cortical atrophy without basal ganglia abnormalities and no clear changes over the 3-year interval. | 80  80  80  49 |
| **ATXN3** | >56 CAG | - | - | 71, 43 |
